# Barriers and Facilitators for Population Genetic Screening in Healthy Populations: A Systematic Review

**DOI:** 10.3389/fgene.2022.865384

**Published:** 2022-07-04

**Authors:** Emily C. Shen, Swetha Srinivasan, Lauren E. Passero, Caitlin G. Allen, Madison Dixon, Kimberly Foss, Brianna Halliburton, Laura V. Milko, Amelia K. Smit, Rebecca Carlson, Megan C. Roberts

**Affiliations:** ^1^ College of Arts and Sciences, University of North Carolina at Chapel Hill, Chapel Hill, NC, United States; ^2^ UNC Lineberger Comprehensive Cancer Center, School of Medicine, University of North Carolina, Chapel Hill, NC, United States; ^3^ Division of Pharmaceutical Outcomes and Policy, Eshelman School of Pharmacy, University of North Carolina, Chapel Hill, NC, United States; ^4^ Department of Public Health Science, College of Medicine, Medical University of South Carolina, Charleston, SC, United States; ^5^ Department of Behavioral, Social, and Health Education Science, Rollins School of Public Health, Emory University, Atlanta, GA, United States; ^6^ Department of Genetics, School of Medicine, University of North Carolina, Chapel Hill, NC, United States; ^7^ The Daffodil Centre, University of Sydney, A Joint Venture with Cancer Council NSW, Sydney, NSW, Australia; ^8^ Melanoma Institute Australia, University of Sydney, Sydney, NSW, Australia; ^9^ Health Sciences Library, University of North Carolina, Chapel Hill, NC, United States

**Keywords:** population testing, universal genetic screening, healthy population screening, average risk, precision public health, perceptions, attitudes, outcomes

## Abstract

Studies suggest that 1–3% of the general population in the United States unknowingly carry a genetic risk factor for a common hereditary disease. Population genetic screening is the process of offering otherwise healthy patients in the general population testing for genomic variants that predispose them to diseases that are clinically actionable, meaning that they can be prevented or mitigated if they are detected early. Population genetic screening may significantly reduce morbidity and mortality from these diseases by informing risk-specific prevention or treatment strategies and facilitating appropriate participation in early detection. To better understand current barriers, facilitators, perceptions, and outcomes related to the implementation of population genetic screening, we conducted a systematic review and searched PubMed, Embase, and Scopus for articles published from date of database inception to May 2020. We included articles that 1) detailed the perspectives of participants in population genetic screening programs and 2) described the barriers, facilitators, perceptions, and outcomes related to population genetic screening programs among patients, healthcare providers, and the public. We excluded articles that 1) focused on direct-to-consumer or risk-based genetic testing and 2) were published before January 2000. Thirty articles met these criteria. Barriers and facilitators to population genetic screening were organized by the Social Ecological Model and further categorized by themes. We found that research in population genetic screening has focused on stakeholder attitudes with all included studies designed to elucidate individuals’ perceptions. Additionally, inadequate knowledge and perceived limited clinical utility presented a barrier for healthcare provider uptake. There were very few studies that conducted long-term follow-up and evaluation of population genetic screening. Our findings suggest that these and other factors, such as prescreen counseling and education, may play a role in the adoption and implementation of population genetic screening. Future studies to investigate macro-level determinants, strategies to increase provider buy-in and knowledge, delivery models for prescreen counseling, and long-term outcomes of population genetic screening are needed for the effective design and implementation of such programs.

Systematic Review Registration: https://www.crd.york.ac.uk/prospero/display_record.php?ID=CRD42020198198

## 1 Introduction

Studies suggest that 1–3% of the general population in the United States carry a genetic risk factor for a common hereditary disease. Typically, genetic testing approaches for identifying these individuals are limited to testing those at high risk of hereditary disease (e.g., cascade testing for at-risk relatives of individuals with a diagnosis). Conversely, population genetic screening offers genetic testing (for common genomic variants) to otherwise healthy individuals to inform risk assessment, precision prevention and early detection of preventable, common diseases. A key example of population genetic screening is newborn screening, which is often celebrated as one of public health’s best accomplishments (Murray et al., 2018).

The Centers for Disease Control and Prevention Office of Genomics and Precision Health has prioritized population genetic screening for common disease conditions (Hereditary Breast and Ovarian Cancer, Lynch Syndrome, and familial hypercholesterolemia) as Tier 1 applications for genomics due to their “significant potential for positive impact on public health” (CDC, 2021). While clinical evidence is currently insufficient to recommend widespread screening in healthy populations (Hampel and de la Chapelle, 2011; Representatives of the Global Familial Hypercholesterolemia Community, 2020), clinical pilot programs are in place to understand cost-efficiency, implementation, and other health related outcomes of population genetic screening (Hay et al., 2021; Lacson et al., 2021; Smit et al., 2021). These pilot studies are on the rise and offer promising opportunities to build the necessary knowledge base for expanding population genetic screening.

Understanding the barriers, facilitators, perceptions, and outcomes to population genetic screening of healthy populations is critical for implementing screening programs in healthcare settings. Previous systematic reviews relating to population genetic screening focus on economic and informed choice evaluations (Rogowski, 2006; Ames et al., 2015). To address this need, we conducted a systematic review of current research literature to understand the barriers, facilitators, perceptions, and outcomes that will be vital for the successful translation of research to support population genetic screening (if found to be appropriate for scaling up).

## 2 Methods

### 2.1 Protocol and Registration

We adhered to the Preferred Reporting Items for Systematic Review and Meta-analyses (PRISMA) reporting guidelines (Moher et al., 2009) for this review (Supplementary Appendix SA). Details of the protocol for this systematic review were registered on PROSPERO and can be accessed at https://www.crd.york.ac.uk/prospero/display_record.php?ID=CRD42020198198 (Shen et al., 2022).

### 2.2 Search Strategy and Information Sources

We worked with a medical librarian (RC) to develop search strategies for the concept of population genetic screening in unknown- and average-risk populations in PubMed, Embase, and Scopus from date of database inception to 22 May 2020, when all searches were completed. Search filters were used to limit the results to original research articles written in English and to exclude preconception, prenatal, and carrier testing. The complete strategy for each of the searches can be found in Supplementary Appendix SB. We also manually examined the references of relevant literature reviews to identify additional studies that may have been missed by the database searches. All references were uploaded to Veritas Health Innovation Covidence systematic review software, 2021 (Veritas Health Innovation), a systematic review management system for study selection.

### 2.3 Eligibility Criteria

Conference abstracts, meeting reports, literature reviews, guidelines, and simulation modeling studies were excluded. Articles focusing on genetic literacy and research, hypothetical gene correlations, and those that lacked a methods section or relevant outcomes were also excluded. Finally, we excluded articles that focused on direct-to-consumer or high-risk genetic testing and articles that were published before 1 January 2000 to understand views of population genetic screening with the use of contemporary technology.

### 2.4 Study Selection

Each title and abstract were reviewed independently for eligibility by random sets of two reviewers (ES, SS, LP, CA, MD, KF, BH, LM, AS) and thematic issues were resolved by discussion. MR oversaw the process and formally resolved specific conflicts. Each full text was assessed independently by random sets of two reviewers (ES, SS, LP, CA, MD, BH, LM, AS) and thematic issues were resolved by discussion. KF oversaw this process and formally resolved specific conflicts. We included articles that detailed the perspectives of participants of population genetic screening programs and individuals asked about population genetic screening to capture all possible barriers, facilitators, perceptions, and outcomes from the position of patients, healthcare providers, and the public.

### 2.5 Data Items and Data Collection Process

Data extraction forms were developed in Covidence using the PICOS framework (Schardt et al., 2007) (see Supplementary Appendix SC) to collect information about each study’s population (patients, healthcare providers, and the public), intervention (disease area(s), whether population genetic screening was offered, and whether participants met with providers before or after screening), comparator group if applicable, outcomes (barriers, facilitators, perceptions, effectiveness measures), and setting (e.g., scale, country, type). We defined patients as healthy individuals with no known risk status who were seen in the healthcare system and the public as individuals who were selected from and represented the broader community. For studies that investigated more than three disease areas, we list their disease areas as “a variety of conditions” for simplicity. We note whether testing for monogenic or polygenic conditions were performed or proposed for consideration by the study. It can be noted that common genomic variants may vary from program to program.

We categorized effectiveness measures as Results (results of the actual screening), Follow-up, Change in Health Behavior, and Interpretation (ex: participants’ emotional responses, risk perception changes, etc.).

The extraction forms were developed based on a previous review (Srinivasan et al., 2020) and four sets of two reviewers independently piloted them on a subset of five articles to agree on a final version. ES, SS, and LP resolved disagreements in data extractions and discussed specific articles as needed. We separately examined articles that had implemented population genetic screening and those that had not implemented population genetic screening to account for contextual differences before analyzing these article types together. Barriers and facilitators were arranged according to the Social Ecological Model (Golden and Earp, 2012), which views health as being affected by interactions at the intrapersonal, interpersonal, and community levels. Perceptions were categorized into favorable, unfavorable, and in-between.

We initially aimed to understand barriers, facilitators, perceptions, and outcomes. It became apparent that barriers and facilitators were related to perceptions, and overall outcomes were quite diverse and hard to summarize across heterogeneous studies, therefore we focus our results on barriers and facilitators.

### 2.6 Risk of Bias in Individual Studies

Reviewers independently assessed the methodological quality of each study following the Mixed Method Appraisal Tool, version 2018 (Hong et al., 2018) for each study type (RCT, descriptive, observation, qualitative, or mixed methods). Meta-analysis was not conducted due to the high variation in study design, population, setting, and outcomes. Due to the small number of studies, we did not define a threshold with which to exclude “low quality” studies. To prevent highlighting any such studies, we ensured that our discussion points were present in multiple studies that mostly have an MMAT score of 3 or higher.

## 3 Results

### 3.1 Study Characteristics

**FIGURE 1:**
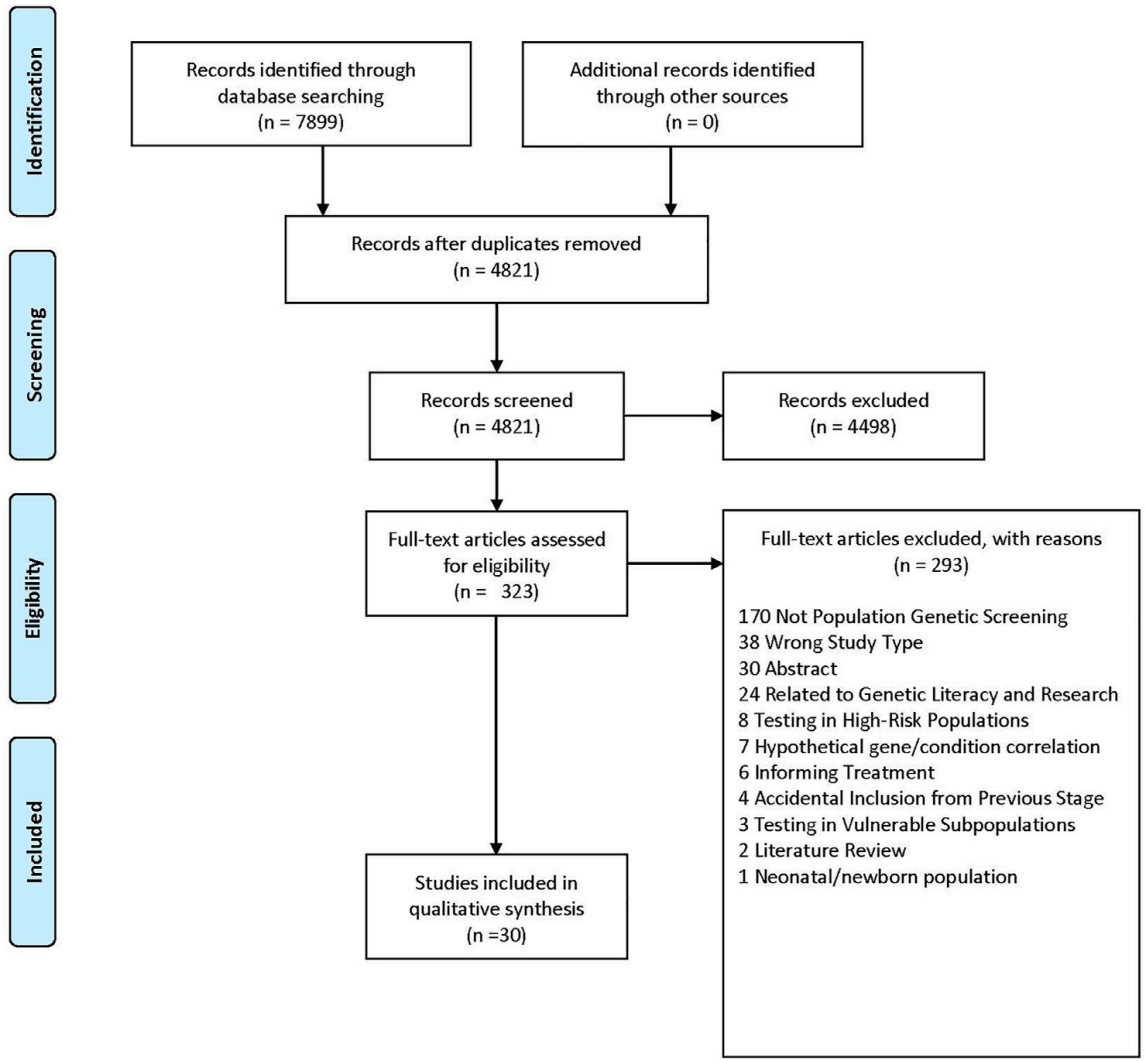
PRISMA diagram.

Characteristics of our included studies can be found in Table 1. Of the 4,821 unique studies that were identified through database searching, 323 articles were assessed for full-text eligibility (see Figure 1 for PRISMA diagram). Thirty articles were included. (Shaw and Bassi, 2001; Laskey et al., 2003; Toiviainen et al., 2003; Sanderson et al., 2004, 2017; Allen et al., 2008; Borry et al., 2008; Neghina and Anghel, 2010; Haga et al., 2011; Hardie, 2011; Henneman et al., 2011; Nielsen and El-Sohemy, 2012; Nusbaum et al., 2013; Haga et al., 2014; Vassy et al., 2014; Hietaranta-Luoma et al., 2015; O’Neill et al., 2015; Shiloh et al., 2015; Godino et al., 2016; Nicholls et al., 2016; Sanderson et al., 2016; Vassy et al., 2017; Fenton et al., 2018; Hay et al., 2018; East et al., 2019; Rego et al., 2019; Rubinsak et al., 2019; Zoltick et al., 2019; Joshi et al., 2020; Smit et al., 2020).

**TABLE 1:** Characteristics of included studies.

Study ID	Setting				Methods					Population					Intervention					
	Year Published	Country	Setting Type	Years of data collection	Scale	Study Design	Data source	Effectiveness Measures Captured	MMAT Score	Types of stakeholders	% Female	Mean Age	% White	Other race or ethnicity information	Disease Areas	Monogenic/Polygenic Condition	Population that genetic screening was offered	Comparison Group	Type of healthcare provider available for prescreen consultation	Type of healthcare provider available for post-screen consultation
Allen et al. (2008)	2008	Australia	Community	NR	City/town	Descriptive	Questionnaire data	Results, Follow-up, Change in Health Behavior, Interpretation	5	Patients	53	41.6	NR	NR	HFE-associated hereditary haemochromatosis	Monogenic	Individuals who worked at workplaces that HaemScreen was implemented	N/A	NR	Physicians
Borry et al. (2008)	2008	European Union	NR	2006–2007	International	Descriptive	Questionnaire data	N/A	4	Providers (Clinical geneticists)	47	NR	NR	NR	A variety of conditions	Monogenic	N/A	N/A	N/A	N/A
East et al. (2019)	2019	United States	Clinic	2015–2018	Single Center	Descriptive	Survey data	N/A	4	Patients	59	40	NR	NR	NR	N/A	Patients seen at the Smith Family Clinic for Genomic Medicine, LLC. categorized as elective (part of the Insight Genome program)	Patients categorized as diagnostic (evaluated because of a personal or family history of disease)	Medical Geneticist & Genetic Counselor	NR
Fenton et al. (2018)	2018	Australia	Community	NR	State	Mixed Methods	Questionnaire	Follow-up	3	Public	50	NR	NR	NR	Melanoma	Polygenic	Individuals 18–69 years old with no personal history of melanoma who are part of the Cancer Council NSW “Join a Research Study” database	N/A	Genetic Counselor	Genetic Counselor
Godino et al. (2016)	2016	United Kingdom	Community	2011	National	RCT	Questionnaire data	Follow-up, Change in Health Behavior, Interpretation	4	Public	53	48.7	NR	NR	Type 2 diabetes mellitus	Polygenic	Individuals born between 1950 and 1975 registered with participating general practices in Cambridgeshire, United Kingdom and enrolled in the Fenland Study	Participants given no risk estimate or phenotypic risk estimate	NR	NR
Haga et al. (2011)	2011	United States	Clinic	2010	National	Descriptive	Survey data	N/A	3	Providers (Primary care)	15	NR	94	0.6% African American, 3.8% Asian, 2.5% other/prefer not to answer, 1.9% Hispanic	A variety of conditions	Polygenic	N/A	N/A	N/A	N/A
Haga et al. (2014)	2014	United States	Clinic	NR	Single Center	RCT	Survey data	Results, Interpretation	2	Public	70	NR	60	22% Black 8% Other 1.7% Prefer not to answer 0.4% Unsure	Type 2 diabetes mellitus	Polygenic	Non-diabetic participants recruited from Duke University (Durham, NC) and surrounding areas	N/A	NR	Genetic Counselor
Hardie, (2011)	2011	Australia	NR	NR	National	Mixed Methods	Survey data	N/A	5	Public	64	54	NR	NR	NR	Polygenic	N/A	N/A	N/A	N/A
Hay et al. (2018)	2018	United States	Clinic	NR	State	RCT	RCT data	N/A	1	Public	79	54	71	48% Hispanic, 3% Black, 3% American Indian/Alaska Native, 2% Asian, 21% Other including Native Hawaiian or multiple races	Melanoma and basal cell carcinoma	Polygenic	Primary care patients 18 years or older at University of New Mexico outpatient primary care clinic	Usual care control	NR	NR
Henneman et al. (2011)	2011	Netherlands	Community	2007	City/town	Qualitative	Focus Group data	N/A	5	Public	100	53.4	92	NR	Breast cancer	Polygenic	N/A	N/A	N/A	N/A
Hietaranta-Luoma et al. (2015)	2015	Finland	Clinic	NR	Regional	RCT	RCT data	Follow-up, Change in Health Behavior, Interpretation	3	Patients	69	47	NR	NR	Cardiovascular disease	Polygenic	Healthy adults aged 20–67 years	Participants who had a session with a nutritionist, received general health and nutrition recommendations, and counseling/lecture by a professor of nutrigenomics	Nutritionist	Medical Doctor
Joshi et al. (2020)	2019	Canada	Clinic	2017–2018	National	Qualitative	Interview data	N/A	5	Providers (Primary care)	NR	NR	NR	NR	NR	Monogenic	N/A	N/A	N/A	N/A
Laskey et al. (2003)	2003	United States	NR	2001	Single Center	Descriptive	Survey data	N/A	4	Public	79	NR	NR	71% African American, 11% Hispanic, 18% listed another race including Filipino, Asian, or Eastern Indian, 0.02% No Response	NR	N/A	N/A	N/A	N/A	N/A
Neghina and Anghel., (2010)	2010	Romania	Clinic	NR	Single Center	Descriptive	Questionnaire data	Results	3	Patients	58	54.8	NR	NR	Hereditary hemochromatosis	Monogenic	Patients 18 years or older who attended the ambulatory unity of the Emergency County Hospital, Timisoara, Romania	N/A	Physician And Health Professional	NR
Nicholls et al. (2016)	2016	Canada	Community	2011–2012	National	Mixed Methods	Written comments, survey, and non-participant observation data	N/A	2	Public	72	58.35	76	1% Native Canadian	Colorectal cancer and type 1 diabetes	Polygenic	N/A	N/A	N/A	N/A
Nielsen and El-Sohemy, (2012)	2012	Canada	Community	2011	National	RCT	Survey data	Interpretation	5	Public	76	26	62	21% East Asian, 11% South Asian, 7% Other	Nutrition	Polygenic	Men and women between the ages of 20–29 years from the Toronto Nutrigenomics and Health Study	Dietary recommendations from health organizations for the same dietary components without genetic information	NR	NR
Nusbaum et al. (2013)	2013	United States	Clinic	NR	Single Center	Qualitative	Interview data	Results, Follow-up, Change in Health Behavior, Interpretation	5	Patients	60	61	65	25% African American, 10% multi-racial	Colorectal cancer	Polygenic	Primary care patients aged 40 and older recruited from the Division of General Internal Medicine at Georgetown University Hospital	N/A	Genetic Counselor	Genetic Counselor
O’Neill et al. (2015)	2015	United States	NR	2007–2008	National	Qualitative	Interview data	Results, Interpretation	4	Public	57	34.89	62	27.63% African American 10.9% Other	A variety of conditions	Polygenic	Participant between 25–40 in the National Human Genome Research Institute’s NHGRI Multiplex Initiative and having no health conditions surveyed through the Multiplex Initiative	N/A	NR	NR
Rego et al. (2019)	2019	United States	Clinic	NR	Single Center	Qualitative	Interview data	Results, Interpretation	5	Public	33	NR	75	NR	A variety of conditions	Both	Adult participants who were recruited from the Integrated Personal Omics Profiling (cohort is enriched for prediabetics)	N/A	NR	Genetic Counselors (Sometimes Included Other Study Team Members: A Medical Geneticist, Neurologist or Endocrinologist, Scientist And/or Student)
Rubinsak et al. (2019)	2019	United States	Clinic	2018	Single Center	Descriptive	Survey data	N/A	3	Patients	100	37.7	37	50.5% Black, 12.1%	Hereditary Breast and Ovarian Cancer	Monogenic	N/A	N/A	N/A	N/A
Sanderson et al. (2004)	2004	United Kingdom	Community	2002	National	Descriptive	Questionnaire data	N/A	4	Public	51	47	94	6% non-Caucasian	Cancer, heart disease	Polygenic	N/A	N/A	N/A	N/A
Sanderson et al. (2016)	2016	United States	Clinic	NR	Single Center	Mixed Methods	Interview and Questionnaire data	Interpretation	2	Public	46	48	71	8.6% African American, 5.7% Hispanic/Latino, 5.7% Asian, 5.7% Multiple Races, 2.9% Self-reported Turkish	A variety of conditions	Both	General population older than 18 at the Mount Sinai Medical Center in New York City	N/A	Genetic Counselor	NR
Sanderson et al. (2017)	2017	United States	Clinic	NR	Single Center	Mixed Methods	Interview and Questionnaire data	Results, Follow-up, Interpretation	1	Public	41	48.6	79	3.4% African American, 3.4% Asian, 6.9% Hispanic/Latino, 6.9% More than 1 race	A variety of conditions	Both	Participants of the HealthSeq project	N/A	Study Genetic Counselor and Medical Geneticist	NR
Shaw and Bassi, (2001)	2001	United States	Community	NR	City/town	Descriptive	Survey data	N/A	2	Public	54	51.8	95	1.8% African American, 0.9% Asian American, 0.9% Native American, and 1.7% Other	NR	Monogenic	N/A	N/A	N/A	N/A
Shiloh et al. (2015)	2014	United States	Clinic	NR	National	Non-RCT	Interviews	Results, Interpretation	2	Public	57	35	NR	38% African American	A variety of conditions	Polygenic	Adults ages 25–40 years old, not affected by Type2 diabetes, heart disease, high cholesterol, high blood pressure, osteoporosis, or lung, colon, or skin cancer	N/A	NR	Research Educator
Smit et al. (2020)	2020	Australia	Community	NR	State	Qualitative	Interview data	N/A	5	Public	50	53	NR	NR	Melanoma	Polygenic	All participants part of a pilot trial to give information on personalized melanoma genomic risk to the public	N/A	Genetic Counselor	NR
Toiviainen et al. (2003)	2003	Finland	Community	1996–1998	National	Descriptive	Survey data	N/A	3	Providers (Gynaecologist, Pediatrician, Clinical geneticist, General practitioner midwife, public health nurse and Public	66	43.5	NR	NR	A variety of conditions	Monogenic	N/A	N/A	N/A	N/A
Vassy et al. (2014)	2015	United States	Clinic	2013	City/town	Mixed Methods	Interview and survey data	N/A	5	Providers (Primary care or Cardiologist)	39	52	78	22.22% Non-white race/ethnicity	NR	Both	N/A	Evaluating patients based on family history only	N/A	N/A
Vassy et al. (2017)	2017	United States	Clinic	NR	City/town	RCT	Survey data	Results, Follow-up, Change in Health Behavior, Interpretation	3	Patients and providers (Primary care)	58	55	89	11% Other	A variety of conditions	Monogenic	Participants (45–60) of the MedSeq Project	N/A	Primary Care provider	Primary Care Provider
Zoltick et al. (2019)	2019	United States	Clinic	2014–2017	National	Descriptive	Survey data	Change in Health Behavior, Interpretation	4	Public	38	53	92	2.8% Asian 0.6% African American/Black 4.9% More than one race/other	A variety of conditions	Monogenic	Adults aged 18 years or older who independently decided to pursue pre-dispositional personal genome sequencing through one of the collaborating projects (PGP, Health-Seq, and the YPO and MD/PhD Genome Projects)	N/A	Varies By Project	Varies By Project

Most studies investigated the perspectives of the public (*n* = 18) (Shaw and Bassi, 2001; Laskey et al., 2003; Sanderson et al., 2004, 2017; Hardie, 2011; Henneman et al., 2011; Nielsen and El-Sohemy, 2012; Haga et al., 2014; O’Neill et al., 2015; Shiloh et al., 2015; Godino et al., 2016; Nicholls et al., 2016; Sanderson et al., 2016; Fenton et al., 2018; Hay et al., 2018; Rego et al., 2019; Zoltick et al., 2019; Smit et al., 2020), while six studies investigated the perspective of patients (Allen et al., 2008; Neghina and Anghel, 2010; Nusbaum et al., 2013; Hietaranta-Luoma et al., 2015; East et al., 2019; Rubinsak et al., 2019), only four investigated the perspective of providers (Borry et al., 2008; Haga et al., 2011; Vassy et al., 2014; Joshi et al., 2020), and two investigated multiple perspectives (Toiviainen et al., 2003; Vassy et al., 2017).

For the most part, studies reported key patient characteristics; however, eleven studies did not record race or ethnicity information (Toiviainen et al., 2003; Allen et al., 2008; Borry et al., 2008; Neghina and Anghel, 2010; Hardie, 2011; Hietaranta-Luoma et al., 2015; Godino et al., 2016; Fenton et al., 2018; East et al., 2019; Joshi et al., 2020; Smit et al., 2020) and one study did not record information about gender or sex (Joshi et al., 2020).

The included studies examined population genetic screening in the context of a variety of conditions, with the most common being melanoma (*n* = 2) (Fenton et al., 2018; Hay et al., 2018; Smit et al., 2020), Type 2 diabetes mellitus (*n* = 2) (Haga et al., 2014; Godino et al., 2016), hereditary haemochromatosis (*n* = 2) (Allen et al., 2008; Neghina and Anghel, 2010), and colorectal cancer (*n* = 2) (Nusbaum et al., 2013; Nicholls et al., 2016).

The majority (*n* = 18) implemented population genetic screening programs of some kind (Allen et al., 2008; Neghina and Anghel, 2010; Nielsen and El-Sohemy, 2012; Nusbaum et al., 2013; Haga et al., 2014; Hietaranta-Luoma et al., 2015; O’Neill et al., 2015; Shiloh et al., 2015; Godino et al., 2016; Sanderson et al., 2016; Sanderson et al., 2017; Vassy et al., 2017; Fenton et al., 2018; Hay et al., 2018; East et al., 2019; Rego et al., 2019; Zoltick et al., 2019; Smit et al., 2020), and the remaining 12 investigated individuals’ opinions on population genetic screening (Shaw and Bassi, 2001; Laskey et al., 2003; Toiviainen et al., 2003; Sanderson et al., 2004; Borry et al., 2008; Haga et al., 2011; Hardie, 2011; Henneman et al., 2011; Vassy et al., 2014; Nicholls et al., 2016; Rubinsak et al., 2019; Joshi et al., 2020).

Of those that implemented screening programs, many utilized genetic counseling either before screening (*n* = 5) (Neghina and Anghel, 2010; Sanderson et al., 2016; Sanderson et al., 2017; East et al., 2019; Smit et al., 2020), after screening (*n* = 4) (Allen et al., 2008; Haga et al., 2014; Shiloh et al., 2015; Rego et al., 2019), or both (*n* = 5) (Nusbaum et al., 2013; Hietaranta-Luoma et al., 2015; Vassy et al., 2017; Fenton et al., 2018; Zoltick et al., 2019). Four did not record counseling availability (Nielsen and El-Sohemy, 2012; O’Neill et al., 2015; Godino. et al., 2016; Hay et al., 2018).

The majority of studies (*n* = 16) were conducted in the US (Shaw and Bassi, 2001; Laskey et al., 2003; Haga et al., 2011; Nusbaum et al., 2013; Haga et al., 2014; Vassy et al., 2014; O’Neill et al., 2015; Shiloh et al., 2015; Sanderson et al., 2016; Sanderson et al., 2017; Vassy et al., 2017; Hay et al., 2018; East et al., 2019; Rego et al., 2019; Rubinsak et al., 2019; Zoltick et al., 2019) and were conducted in a clinical setting (*n* = 16) (Neghina and Anghel, 2010; Haga et al., 2011; Nusbaum et al., 2013; Haga et al., 2014; Vassy et al., 2014; Hietaranta-Luoma et al., 2015; Shiloh et al., 2015; Sanderson et al., 2016; Sanderson et al., 2017; Vassy et al., 2017; Hay et al., 2018; East et al., 2019; Rego et al., 2019; Rubinsak et al., 2019; Zoltick et al., 2019; Joshi et al., 2020) or the community setting (*n* = 10) (Shaw and Bassi, 2001; Toiviainen et al., 2003; Sanderson et al., 2004; Allen et al., 2008; Henneman et al., 2011; Nielsen and El-Sohemy, 2012; Godino et al., 2016; Nicholls et al., 2016; Fenton et al., 2018; Smit et al., 2020).

Included studies included a variety of study designs and received a range of MMAT scores. Of note, 23 studies received an MMAT score of 3 or greater (Laskey et al., 2003; Toiviainen et al., 2003; Sanderson et al., 2004; Allen et al., 2008; Borry et al., 2008; Neghina and Anghel, 2010; Hardie, 2011; Henneman et al., 2011; Nielsen and El-Sohemy, 2012; Nusbaum et al., 2013; Haga et al., 2014; Vassy et al., 2014; Hietaranta-Luoma et al., 2015; O’Neill et al., 2015; Godino et al., 2016; Vassy et al., 2017; Fenton et al., 2018; East et al., 2019; Rego et al., 2019; Rubinsak et al., 2019; Zoltick et al., 2019; Joshi et al., 2020; Smit et al., 2020), and only seven studies received an MMAT score below 3 (Shaw and Bassi, 2001; Haga et al., 2014; Shiloh et al., 2015; Nicholls et al., 2016; Sanderson et al., 2016; Sanderson et al., 2017; Hay et al., 2018).

### 3.2 Barriers

Intrapersonal, interpersonal, and community barriers are reported in Table 2 and below.

**TABLE 2:** Barriers to interest and participation in population genetic screening.

Reasons	Patient				Provider				Public			
	N	%	Significance	Study	N	%	Significance	Study	N	%	Significance	Study
**Intrapersonal**												
Psychosocial Factors, Knowledge, Attitudes, and Beliefs												
Anxiety, fear, and worry toward screening				Nusbaum et al. (2013); Rubinsak et al. (2019)								Hardie, (2011)
Potential negative psychological and emotional impacts								Joshi et al. (2020)	18	50		Sanderson et al. (2016)
												Henneman et al. (2011)
Mistrust												Hardie, (2011)
Possibility of unwanted information												Zoltick et al. (2019)
Belief that low risk result may not give reassurance												Henneman et al. (2011)
Inadequate knowledge						41		Haga et al. (2011)				
								Joshi et al. (2020)				
Not having ordered a genetic test for themselves								Haga et al. (2011)				
Belief that it would not provide useful information						36		Haga et al. (2011)				
Dislike of blood		11		Neghina and Anghel., (2010)								
Moral and ethical reasons												Shaw and Bassi (2001); Hardie (2011)
Disinterest		18.5		Neghina and Anghel., (2010)								Hardie, (2011)
Belief that it would lead unnecessary testing								Vassy et al. (2014)				
Lack of information		41		Neghina and Anghel., (2010)								
				Nusbaum et al. (2013); Rubinsak et al. (2019)								
**Clinical Factors**												
Uncertainty of results								Vassy et al. (2014); Joshi et al. (2020)				Zoltick et al. (2019)
Limited clinical utility								(Borry et al. (2008); Vassy et al. (2014); Joshi et al. (2020)				
**Other**												
Cost				Rubinsak et al. (2019)								Hardie (2011); Zoltick et al. (2019)
Lack of time		32.5		(Neghina and Anghel (2010), 201)								
Higher education												Sanderson et al. (2004)
Religious reasons												Hardie (2011)
**Interpersonal Barriers**												
Family												
Impact on children												Sanderson et al. (2016)
Lack of family history				Rubinsak et al. (2019)								Hardie, (2011)
**Community**												
Data												
Confidentiality/privacy				Nusbaum et al. (2013)		43		Haga et al. (2011)	20	57		Sanderson et al. (2016)
												Zoltick et al. (2019)
Data security								Joshi et al. (2020)				
**Healthcare System**												
Potential impact on insurance						50		Haga et al. (2011)				Henneman et al. (2011); Zoltick et al. (2019)
								Joshi et al. (2020)				
Cost to health system								Joshi et al. (2020)				Henneman et al. (2011); Smit et al. (2020)
**Other**												
Possibility for discrimination by employers								Joshi et al. (2020)				Henneman et al. (2011)

Select studies report the count of participants who agree with facilitator statement (which we label as column “N”), the percentage of participants (which we label as column “%”), and significance levels of the statements (which we label as column “Significance”).

#### 3.2.1 Intrapersonal Barriers

##### 3.2.1.1 Psychosocial Factors, Knowledge, Attitudes, and Beliefs

Psychosocial factors such as anxiety, fear, and worry about screening (Hardie, 2011; Nusbaum et al., 2013; Rubinsak et al., 2019), dislike of blood (Neghina and Anghel, 2010), and potential negative psychological and emotional impacts (Henneman et al., 2011; Sanderson et al., 2016; Joshi et al., 2020) were reported as reasons to reject screening. Additional factors such as mistrust (Hardie, 2011), disinterest (Neghina and Anghel, 2010; Hardie, 2011), the possibility of receiving unwanted information (Zoltick et al., 2019), and the belief that a low-risk result may not give reassurance (Henneman et al., 2011) were reported barriers.

Two studies reported moral and ethical reasons, such as the fear of eugenics and a question of human mortality, as barriers (Shaw and Bassi, 2001; Hardie, 2011). Providers cited inadequate knowledge (Haga et al., 2011; Joshi et al., 2020), not having ordered a genetic test for themselves (Haga et al., 2011), their belief that it would not provide useful information (Haga et al., 2011), and their belief that it would lead to unnecessary future testing (Vassy et al., 2014) as barriers to participating in population genetic screening programs. Additionally, patients reported a lack of information about these programs (Neghina and Anghel, 2010; Nusbaum et al., 2013; Rubinsak et al., 2019).

##### 3.2.1.2 Clinical Factors

Providers (Vassy et al., 2014; Joshi et al., 2020) and the public (Zoltick et al., 2019) cited the uncertainty of results as a barrier for interest and/or participation in screening programs with providers additionally reporting perceived limited clinical utility (Borry et al., 2008; Vassy et al., 2014; Joshi et al., 2020).

##### 3.2.1.3 Other

Perceived cost of population genetic screening (Hardie, 2011; Rubinsak et al., 2019; Zoltick et al., 2019), religious reasons (Hardie, 2011), and higher education (Sanderson et al., 2004) among patients and the public were reported as other barriers for interest and/or participation as well as a lack of time (Neghina and Anghel, 2010).

#### 3.2.2 Interpersonal Barriers

##### 3.2.2.1 Family

A perceived potential for a negative impact on children (Sanderson et al., 2016) and a lack of family history (Hardie, 2011; Rubinsak et al., 2019) were negatively associated with interest and/or participation of population genetic screening among patients and the public.

#### 3.2.3 Community Barriers

##### 3.2.3.1 Data

Concerns related to confidentiality and privacy (Haga et al., 2011; Nusbaum et al., 2013; Sanderson et al., 2016; Zoltick et al., 2019) and data security (Joshi et al., 2020) were reported as barriers across stakeholders.

##### 3.2.3.2 Healthcare System

Providers and the public reported that the potential impact of results on insurance (Haga et al., 2011; Henneman et al., 2011; Zoltick et al., 2019; Joshi et al., 2020) and the potential increased cost to the health system (Henneman et al., 2011; Joshi et al., 2020; Smit et al., 2020) would hinder their participation in population genetic screening.

##### 3.2.3.3 Other

The possibility for discrimination by employers was reported by providers and the public (Henneman et al., 2011; Joshi et al., 2020).

### 3.3 Facilitators

Intrapersonal, interpersonal, and community facilitators can be found in Table 3 and below.

**TABLE 3:** Facilitators to interest and participation in population genetic screening.

Reasons	Patient				Provider				Public			
	N	%	Significance	Study	N	%	Significance	Study	N	%	Significance	Study
**Intrapersona**l												
**Demographics and Socio-Economic Status**												
Male gender										72	*p* = 0.029	Sanderson et al. (2004)
Later middle age										78		Sanderson et al. (2004)
Younger age				Neghina and Anghel., (2010)								
Higher socio-economic status				Neghina and Anghel., (2010)								Hay et al. (2018)
**Psychosocial Factors, Knowledge, Attitudes, and Beliefs**												
Interest about ancestry									13			Sanderson et al. (2016)
												Zoltick et al. (2019)
Professional interest/utility									1			Sanderson et al. (2016)
												Zoltick et al. (2019)
Interest in genetics/science												Sanderson et al. (2016); Rego et al. (2019); Zoltick et al. (2019)
General curiosity				Nusbaum et al. (2013); East et al. (2019)								Hardie (2011); Zoltick et al. (2019)
										66		Sanderson et al. (2016)
Chance to learn about themselves				Rubinsak et al. (2019)						86		Nielsen and El-Sohemy, (2012)
									7			Sanderson et al. (2016)
Altruism				Nusbaum et al. (2013)								Rego et al. (2019)
									15			Sanderson et al. (2016)
Trust in provider											*p* < 0.001	Hardie, (2011)
Trust in medicine											*p* < 0.001	Hardie, (2011)
Belief that screening will yield helpful information												Shaw and Bassi, (2001)
Knowledge								Borry et al. (2008); Haga et al. (2011)				
Nothing to lose				Nusbaum et al. (2013)								
Chance to have a free screen		71.4		Neghina and Anghel., (2010)								
Novel opportunity												Sanderson et al. (2016)
Fun and entertaining												Zoltick et al. (2019)
**Clinical Factors**												
Known or suspected personal history												Sanderson et al. (2016); Hay et al. (2018)
Curability of condition											*p* < 0.001	Shaw and Bassi, (2001)
More certain outcome												Shaw and Bassi, (2001)
Non-fatalness of condition											*p* < 0.01	Shaw and Bassi, (2001)
Prepare for future health		57		East et al. (2019)								Nicholls et al. (2016); Sanderson et al. (2016); Rego et al. (2019); Zoltick et al. (2019)
Potential for medical intervention/monitoring				East et al. (2019)				Borry et al. (2008); Joshi et al. (2020)		73		Nielsen and El-Sohemy, (2012)
												Sanderson et al. (2016)
Potential to encourage health improvements												Hardie (2011); Sanderson et al. (2016); Zoltick et al. (2019)
										83		Nielsen and El-Sohemy, (2012)
Seeking medical information		37		East et al. (2019)								
		85.7		Neghina and Anghel., (2010)								
				Nusbaum et al. (2013)								
Diagnostic purposes									1			Sanderson et al. (2016)
Pharmacogenomics				East et al. (2019)								Sanderson et al. (2016); Zoltick et al. (2019)
**Interpersonal**												
**Family**												
Provide information for family members		40		East et al. (2019)								Nicholls et al. (2016); Rego et al. (2019); Zoltick et al. (2019)
				Nusbaum et al. (2013); Rubinsak et al. (2019)					11			Sanderson et al. (2016)
Having family who have had their genomes sequenced												Zoltick et al. (2019)
Family history				Rubinsak et al. (2019)								Hardie (2011); Hay et al. (2018); Rego et al. (2019); Zoltick et al. (2019)
										74	*p* = 0.005	Sanderson et al. (2004)
										33		Sanderson et al. (2016)
Lack of family health history									1			Rego et al. (2019)
										70		Sanderson et al. (2004)
												Sanderson et al. (2016); Zoltick et al. (2019)

#### 3.3.1 Intrapersonal Facilitators

##### 3.3.1.1 Demographics and Socio-Economic Status

One study (Sanderson et al., 2004) reported that male gender (*p* = 0.029) and later middle age were positively correlated with an interest in screening. On the other hand, another study (Neghina and Anghel, 2010) reported that younger age was a facilitator to uptake of screening. Higher socioeconomic status was additionally cited as a facilitator to participation (Neghina and Anghel, 2010; Hay et al., 2018).

##### 3.3.1.2 Psychosocial Factors, Knowledge, Attitudes, and Beliefs

Attitudes related to having an interest about ancestry (Sanderson et al., 2016; Zoltick et al., 2019), professional interest (Sanderson et al., 2016; Zoltick et al., 2019), interest in genetics and/or science (Sanderson et al., 2016; Rego et al., 2019; Zoltick et al., 2019), and general curiosity (Hardie, 2011; Nusbaum et al., 2013; Sanderson et al., 2016; East et al., 2019; Zoltick et al., 2019) were reported facilitators for screening. Additional facilitators include altruism (Nusbaum et al., 2013; Sanderson et al., 2016; Rego et al., 2019) and the chance for participants to learn about themselves (Nielsen and El-Sohemy, 2012; Sanderson et al., 2016; Rubinsak et al., 2019).

Knowledge (Borry et al., 2008; Haga et al., 2011), the belief that screening will provide helpful information (Shaw and Bassi, 2001), trust in provider (Hardie, 2011) and trust in medicine (Hardie, 2011) were all associated with interest in population genetic screening, with the latter two being statistically significant.

Patients reported that the chance to have a free screen (Neghina and Anghel, 2010) and a “nothing to lose” attitude (Nusbaum et al., 2013) and the public reported that viewing population genetic screening as a novel opportunity (Sanderson et al., 2016) and a fun and entertaining activity (Zoltick et al., 2019) were facilitators for undergoing screening.

##### 3.3.1.3 Clinical Factors

All stakeholders viewed the potential for medical intervention and/or monitoring (Borry et al., 2008; Nielsen and El-Sohemy, 2012; Sanderson et al., 2016; East et al., 2019; Joshi et al., 2020) as a facilitator to population genetic screening. The public reported that curability (*p* < 0.001) (Shaw and Bassi, 2001), non-fatalness of a condition (*p* < 0.01) (Shaw and Bassi, 2001), a more certain outcome (Shaw and Bassi, 2001), a known or suspected personal history (Sanderson et al., 2016; Hay et al., 2018), the potential to encourage health improvements through means such as behavioral changes (Hardie, 2011; Nielsen and El-Sohemy, 2012; Sanderson et al., 2016; Zoltick et al., 2019), and the use of results for future diagnostic purposes (Sanderson et al., 2016) were positively associated with interest and/or receipt of population genetic screening through a population-based context.

Additionally, patients reported their seeking medical information as a reason for receiving screening (Neghina and Anghel., 2010; Nusbaum et al., 2013; East et al., 2019). Patients and the public reported that the ability to prepare for future health (Nicholls et al., 2016; Sanderson et al., 2016; East et al., 2019; Rego et al., 2019; Zoltick et al., 2019) and the use of results for pharmacogenomics (Sanderson et al., 2016; East et al., 2019; Zoltick et al., 2019) were facilitators to population genetic screening.

#### 3.3.2 Interpersonal Facilitators

##### 3.3.2.1 Family

All interpersonal facilitators were related to participants’ family. Patients and the public reported that the ability to provide information to family members to them (Nusbaum et al., 2013; Nicholls et al., 2016; Sanderson et al., 2016; East et al., 2019; Rego et al., 2019; Rubinsak et al., 2019; Zoltick et al., 2019). Having family who have had their genomes sequenced facilitated participation as well (Zoltick et al., 2019).

Family history positively associated with both interest and/or participation in population genetic screening (Hardie, 2011; Sanderson et al., 2016; Hay et al., 2018; Rego et al., 2019; Rubinsak et al., 2019; Zoltick et al., 2019) and labeled as a statistically significant factor in one study (Sanderson et al., 2004). On the other hand, a lack of family health history was also reported as a facilitator for both interest and/or participation in four studies (Sanderson et al., 2004; Sanderson et al., 2016; Rego et al., 2019; Zoltick et al., 2019).

### 3.4 Perceptions

Perceptions are summarized in Supplementary Appendix SD.

### 3.5 Effectiveness Measures

Effectiveness measures are summarized in Supplementary Appendix SE.

## 4 Discussion

Overall, we identified multilevel barriers and facilitators for population genetic screening implementation. Psychosocial and attitudinal barriers, such as anxiety and worry toward screening and the possibility for negative psychological and emotional impacts, were the most reported individual-level barriers across stakeholders, even though studies to date have demonstrated limited impacts on psychological and emotional outcomes with any adverse responses dissipating over time (Hietaranta-Luoma et al., 2015; Hollands et al., 2016; Frieser et al., 2018; Smit et al., 2020).

Skeptical healthcare providers cited a perceived lack of clinical utility as a barrier, reporting that although they believe population genetic screening is valuable, they do not believe that it is ready for clinical use (Joshi et al., 2020). On the other hand, healthcare providers who supported population genetic screening reported the potential for results to inform medical intervention and/or monitoring as a reason for their support. Our findings are consistent with previous literature indicating that obtaining provider buy-in is needed for the implementation of large-scale screening (Peterson et al., 2016). Additionally, the current perception of clinical utility places value on genomic medicine in relation to informing treatment, and excludes other applications for screening such as risk prediction and prognosis (Joseph et al., 2016). The Association for Molecular Pathology (Joseph et al., 2016) recommends expanding the definition of clinical utility for molecular tools through approaches such as utilizing a modified ACCE model (CDC, 2019) and promoting patient-centered definitions of clinical utility. Our data suggests the need for interventions directed toward obtaining buy-in and expanding the definition of clinical utility to include the context of population genetic screening.

Studies also reported potential ethical issues, concerns relating to data management, and potential discrimination as barriers to interest in population genetic screening. These factors are especially important in the age of “big data” (Price and Cohen, 2019), and previous literature has called for the consideration of ethical questions in implementing population genetic screening (Murray et al., 2018). The BabySeq Project is assessing ethical, legal, and social implications (ELSI) relating to the ethical issues of result return (Friedman et al., 2017) and the medical, behavioral, and economic impacts (Holm et al., 2018) of newborn screening. These studies, along with essential ELSI questions raised by newborn screening (Goldenberg et al., 2019), may provide a potential framework that can be adapted for assessing ELSI considerations in evaluating general population genetic screening.

Many of our included studies investigated the general public’s perspective of population genetic screening. This presents an opportunity to focus on the roles of other stakeholders within the larger societal systems, such as healthcare providers and public health officials. Primary care providers, who will likely be the touchpoint for many interested in population genetic screening, reported inadequate knowledge as a barrier to ordering screening. In one study (Haga et al., 2011), roughly half of providers reported that they felt prepared to order population genetic screening. Previous literature has noted the limited evidence regarding the views and roles of healthcare providers in genomic medicine (Hann et al., 2017a; Hauser et al., 2018; Crellin et al., 2019), identified the importance of educational resources for provider preparedness to order and interpret results (Rohrer Vitek et al., 2017; Hauser et al., 2018; Smit et al., 2019), and described the integral role that public health officials will play in insuring proper implementation of population genetic screening (Molster et al., 2018). With few provider-based studies (most of which studied primary care providers) and no public health-based studies, we see a need for increased studies to investigate the viewpoints of these providers and develop the necessary educational interventions.

Furthermore, the current state of research in population genetic screening focuses on individuals, with most studies revealing barriers and facilitators to interest and/or participation in population genetic screening at an individual level. We identified few interpersonal facilitators and barriers and no community-level facilitators. All our included studies were designed to elucidate stakeholders’ views and attitudes. This leaves a large gap in the literature in understanding the complex interactions between communities, the healthcare system, and the public health system. The studies which revealed interpersonal and community factors conducted surveys or semi-structured interviews, suggesting a need for additional studies to explicitly investigate macro-level determinants for population genetic screening that are suited to quantitative methods.

Most (all but two) were conducted in racially/ethnically diverse countries (Australia, Canada, United States, and United Kingdom), however roughly one third did not include information on the race or ethnicity of individuals receiving population genetic screening. This is of particular importance as studies have found ethnic minorities to be generally more apprehensive toward genetic testing than white individuals (Hann et al., 2017b). Without data on race and ethnicity of study populations the generalizability of findings is unclear and we remain unable to monitor disparities in access to population genetic screening. This suggests a need for improved reporting of race/ethnicity in population genetic screening research and a need to focus on health equity.

In addition to this challenge, more general agreement on the terminology and reporting of race, ethnicity, and ancestry in genomic research with an eye toward reproducible, ethical, and equitable research is warranted (Flanagin et al., 2021). Though the National Human Genome Research Institute (NHGRI) boldly predicts that “research in human genomics will have moved beyond population descriptors based on historic social constructs such as race” by 2030 (Green et al., 2020), there are currently numerous challenges inherent in standardizing the use (or disuse) of race and ethnicity and other population descriptors in clinical genetics. Fortunately, the National Academies of Sciences, Engineering, and Medicine established a multi-disciplinary committee to examine the current use of population descriptors in genomics research and identify best practices for improving the use of the terminology in the future.

Many studies incorporated genetic counseling; however, they had varying forms of preintervention information content and delivery and only a few assessed the efficacy of different delivery methods. The best approach and timing for genetic counseling delivery has not yet been determined. To date, there is some evidence showing that different contexts will likely have different requirements (Evans and Manchanda, 2020). For example, while this review explicitly excluded reproductive genetic testing, population-wide screening will nonetheless have profound implications for individuals of reproductive age who would be at risk of passing a hereditary predisposition for a life-threatening condition to existing or future children. This provides an opportunity to implement studies specifically designed to investigate the best manner of prescreen education and counseling specific to the delivery context, such as health literacy levels, cultural considerations, reproductive age, and disease type.

Finally, out of the studies that implemented population genetic screening and collected post-intervention data, only one followed participants for more than 12 months (Allen et al., 2008). Without sufficient long-term data, it is difficult to assess the efficacy of the screening programs at the population level. There is a need for prospective cohort studies and randomized controlled trials to evaluate any long-term benefits, such as clinical and economic outcomes, to population-level genetic screening implementation (Murray et al., 2018, 2020). The BabySeq project provides a model for identifying these long-term outcomes (Holm et al., 2018), which may be adapted to the context of population genetic screening. Such studies will likely address our previous points of determining ELSI factors to population genetic screening and assessing the effects of prescreen education methods as well.

## 5 Limitations

There is a potential for bias as we reported missing items as “not reported” and did not contact authors for additional information. Articles varied as to which outcome was reported (barrier, facilitator, perception, and/or outcome), so some articles may be more represented than others. Our included studies did not assess effect sizes of barriers and facilitators on interest and/or uptake of population genetic screening, which prevented us from conducting a meta-analysis. Additionally, the heterogeneity in disease states and reported effectiveness measures prevented us from fully synthesizing the data. With all systematic reviews, there is the possibility that we missed relevant literature.

## 6 Conclusion

We found that 1) psychosocial, attitudinal, and belief-related factors present a barrier for stakeholders to participate in screening, 2) perceived limited clinical utility presents a barrier for provider uptake, 3) there is a need for additional studies investigating healthcare and public health provider roles and education, 4) research in population genetic screening has focused on stakeholder attitudes, and 5) there is a need for long-term follow-up studies and health equity-focused studies of population genetic screening. Future research should 1) evaluate the best manner for prescreen education and counseling for specific contexts, 2) examine provider buy-in and clinical utility expansion, 3) investigate the views of providers and develop educational resources, 4) investigate macro-level determinants of and address ELSI questions toward population genetic screening, and 5) assess the long-term outcomes of population genetic screening. Taken together this data can inform future interventions to improve the development and implementation of population genetic screening.

## Data Availability

The original contributions presented in the study are included in the article/Supplementary Material, further inquiries can be directed to the corresponding author.
