# Supplementary material for: Barriers and Facilitators for Population Genetic Screening in Healthy Populations: A Systematic Review
Source: Front Genet. 2022 Jul 4;13:865384. doi: 10.3389/fgene.2022.865384 (PMC9289280; doi:10.3389/fgene.2022.865384)
Supplement: Supplementary file 2 [file Table2.DOCX]

**Appendix B:** Electronic search strategies

Total Search Results: 7,899

Total Original Citations: 4,821

PubMed Search Strategy: 2,054 Results

| Search Concept | Search Number | Search Terms |
| --- | --- | --- |
| Genetic Testing | 1 | (Genetic Testing[Mesh] OR Whole Genome Sequencing[Mesh] OR Genetic-testing[tw] OR Genetic-test[tw] OR Genetic-tests[tw] OR Genetic-screen[tw] OR Genetic-screens[tw] OR Genetic-screening[tw] OR Genetic-screenings[tw] OR genotype-testing[tw] OR genotype-screening[tw] OR human-genome-testing[tw] OR human-genome-screening[tw] OR “whole genome sequencing”[tw] OR “whole exome sequencing”[tw] OR “whole-genome-testing”[tw] OR genomic-testing[tiab] OR genomic-sequencing[tiab]) |
| Populations | 2 | (healthy[tiab] OR Universal[tiab] OR Low-risk[tiab] OR lower-risk[tiab] OR average-risk[tiab] OR Unknown-risk[tiab] OR No-family-history[tiab] OR National[tiab] OR country-wide[tiab] OR state-wide[tiab] OR public[tiab] OR “rural population”[tiab] or “suburban population”[tiab] or “urban population”[tiab] OR presymptomatic[tiab] OR asymptomatic[tiab] OR "Rural Population"[Mesh] OR "Suburban Population"[Mesh] OR "Urban Population"[Mesh]) |
| Barriers, Facilitators and Outcomes | 3 | (Public Opinion[mesh] OR Patient Acceptance of Health Care[mesh] OR Intention[mesh] OR Health Knowledge, Attitudes, Practice[mesh] OR Genetic Counseling[Mesh] OR knowledge[tiab] OR knowledgable[tiab] OR attitude[tiab] OR attitudes[tiab] OR intention[tiab] OR intentions[tiab] OR decision-making[tiab] OR barrier[tiab] OR barriers[tiab] OR opinion[tiab] OR opinions[tiab] OR acceptance[tiab] OR perception[tiab] OR perceptions[tiab] OR perceived[tiab] OR facilitate[tiab] OR facilitator[tiab] OR facilitators[tiab] OR motivation[tiab] OR motivations[tiab] OR education[tiab] OR educate[tiab] OR educated[tiab] OR counseling[tiab] OR counsel[tiab] OR counseled[tiab]) |
| Language | 4 | English[language] |
| Publication Type | 5 | ("Book Reviews" [Publication Type] OR "Comment" [Publication Type] OR "Editorial" [Publication Type] OR "Letter" [Publication Type] OR "Meeting Abstracts" [Publication Type] OR "Newspaper Article" [Publication Type] OR "Retracted Publication" [Publication Type] OR "Case Reports" [Publication Type] OR "Case Report"[ti] OR meta analysis[Publication Type] OR systematic review[Publication Type] OR review[Publication Type] OR guideline[Publication Type] OR practice guideline[Publication Type] OR systematic review[ti] OR meta-analysis[ti] OR meta analysis[ti] OR scoping review[ti] OR literature review[ti]) |
| Preconception and Prenatal Studies | 6 | (Microbiota[mesh] OR In Vitro Techniques[mesh] OR Fertilization in Vitro[mesh] OR Preconception Care[mesh] OR Noninvasive Prenatal Testing[mesh] OR Prenatal Diagnosis[mesh] OR Infant, Newborn[mesh] OR Infant, Newborn, Diseases[mesh]) |
| Animal Studies | 7 | (animals[mesh] NOT humans[mesh]) |
| Search Logic | 8 | (1 AND 2 AND 3 AND 4) NOT (5 OR 6 OR 7) |

Embase Search Strategy: 2,410 Results

| Search Concept | Search Number | Search Terms |
| --- | --- | --- |
| Genetic Testing | 1 | ('genetic screening'/exp OR 'genome sequencing'/exp OR Genetic-testing:ti,ab,de,tn OR Genetic-test:ti,ab,de,tn OR Genetic-tests:ti,ab,de,tn OR Genetic-screen:ti,ab,de,tn OR Genetic-screens:ti,ab,de,tn OR Genetic-screening:ti,ab,de,tn OR Genetic-screenings:ti,ab,de,tn OR genotype-testing:ti,ab,de,tn OR genotype-screening:ti,ab,de,tn OR human-genome-testing:ti,ab,de,tn OR human-genome-screening:ti,ab,de,tn OR "whole genome sequencing":ti,ab,de,tn OR "whole exome sequencing":ti,ab,de,tn OR whole-genome-testing:ti,ab,de,tn OR genomic-testing:ti,ab OR genomic-sequencing:ti,ab) |
| Populations | 2 | (healthy:ti,ab OR Universal:ti,ab OR Low-risk:ti,ab OR lower-risk:ti,ab OR average-risk:ti,ab OR Unknown-risk:ti,ab OR No-family-history:ti,ab OR National:ti,ab OR country-wide:ti,ab OR state-wide:ti,ab OR public:ti,ab OR "rural population":ti,ab OR "suburban population":ti,ab OR "urban population":ti,ab OR presymptomatic:ti,ab OR asymptomatic:ti,ab OR 'rural population'/exp OR 'suburban population'/exp OR 'urban population'/exp) |
| Barriers, Facilitators and Outcomes | 3 | ('public opinion'/exp OR 'patient attitude'/exp OR 'intentionality'/exp OR 'attitude to health'/exp OR 'genetic counseling'/exp OR knowledge:ti,ab OR knowledgable:ti,ab OR attitude:ti,ab OR attitudes:ti,ab OR intention:ti,ab OR intentions:ti,ab OR decision-making:ti,ab OR barrier:ti,ab OR barriers:ti,ab OR opinion:ti,ab OR opinions:ti,ab OR acceptance:ti,ab OR perception:ti,ab OR perceptions:ti,ab OR perceived:ti,ab OR facilitate:ti,ab OR facilitator:ti,ab OR facilitators:ti,ab OR motivation:ti,ab OR motivations:ti,ab OR education:ti,ab OR educate:ti,ab OR educated:ti,ab OR counseling:ti,ab OR counsel:ti,ab OR counseled:ti,ab) |
| Language | 4 | English:la |
| Publication Type | 5 | ('abstract report'/exp OR 'book'/exp OR 'conference abstract'/exp OR 'editorial'/exp OR 'letter'/exp OR 'retraction notice'/exp OR 'case report'/exp OR 'meta analysis'/exp OR 'systematic review'/exp OR 'review'/exp OR 'practice guideline'/de OR "Case Report":ti OR "meta analysis":ti OR "scoping review":ti OR "literature review":ti OR "systematic review":ti) |
| Preconception and Prenatal Studies | 6 | ('microflora'/exp OR 'in vitro fertilization'/exp OR 'prepregnancy care'/exp OR 'prenatal diagnosis'/exp OR 'prenatal screening'/exp OR 'infant disease'/exp OR 'infant disease'/exp) |
| Animal Studies | 7 | ('animal'/exp NOT 'human'/exp) |
| Search Logic | 8 | (1 AND 2 AND 3 AND 4) NOT (5 OR 6 OR 7) |

Scopus Search Strategy: 3,435 Results

| Search Concept | Search Number | Search Terms |
| --- | --- | --- |
| Genetic Testing | 1 | (TITLE-ABS-KEY("Genetic-testing") OR TITLE-ABS-KEY("Genetic-test") OR TITLE-ABS-KEY("Genetic-tests") OR TITLE-ABS-KEY("Genetic-screen") OR TITLE-ABS-KEY("Genetic-screens") OR TITLE-ABS-KEY("Genetic-screening") OR TITLE-ABS-KEY("Genetic-screenings") OR TITLE-ABS-KEY("genotype-testing") OR TITLE-ABS-KEY("genotype-screening") OR TITLE-ABS-KEY("human-genome-testing") OR TITLE-ABS-KEY("human-genome-screening") OR TITLE-ABS-KEY("whole genome sequencing") OR TITLE-ABS-KEY("whole exome sequencing") OR TITLE-ABS-KEY("whole-genome-testing") OR TITLE-ABS("genomic-testing") OR TITLE-ABS("genomic-sequencing")) |
| Populations | 2 | (TITLE-ABS("healthy") OR TITLE-ABS("Universal") OR TITLE-ABS("Low-risk") OR TITLE-ABS("lower-risk") OR TITLE-ABS("average-risk") OR TITLE-ABS("Unknown-risk") OR TITLE-ABS("No-family-history") OR TITLE-ABS("National") OR TITLE-ABS("country-wide") OR TITLE-ABS("state-wide") OR TITLE-ABS("public") OR TITLE-ABS("rural population") OR TITLE-ABS("suburban population") OR TITLE-ABS("urban population") OR TITLE-ABS("presymptomatic") OR TITLE-ABS("asymptomatic")) |
| Barriers, Facilitators and Outcomes | 3 | (TITLE-ABS("knowledge") OR TITLE-ABS("knowledgable") OR TITLE-ABS("attitude") OR TITLE-ABS("attitudes") OR TITLE-ABS("intention") OR TITLE-ABS("intentions") OR TITLE-ABS("decision-making") OR TITLE-ABS("barrier") OR TITLE-ABS("barriers") OR TITLE-ABS("opinion") OR TITLE-ABS("opinions") OR TITLE-ABS("acceptance") OR TITLE-ABS("perception") OR TITLE-ABS("perceptions") OR TITLE-ABS("perceived") OR TITLE-ABS("facilitate") OR TITLE-ABS("facilitator") OR TITLE-ABS("facilitators") OR TITLE-ABS("motivation") OR TITLE-ABS("motivations") OR TITLE-ABS("education") OR TITLE-ABS("educate") OR TITLE-ABS("educated") OR TITLE-ABS("counseling") OR TITLE-ABS("counsel") OR TITLE-ABS("counseled")) |
| Language | 4 | LANGUAGE("English") |
| Publication Type | 5 | (DOCTYPE , "ch") OR (DOCTYPE , "no") OR (DOCTYPE , "bk") OR (DOCTYPE , "le") OR (TITLE("Case Report") OR TITLE("meta analysis") OR TITLE("review")) |
| Preconception and Prenatal Studies | 6 | (INDEXTERMS("Microbiota") OR INDEXTERMS("In Vitro Techniques") OR INDEXTERMS("Fertilization in Vitro") OR INDEXTERMS("Preconception Care") OR INDEXTERMS("Noninvasive Prenatal Testing") OR INDEXTERMS("Prenatal Diagnosis") OR INDEXTERMS("Infant")) |
| Animal Studies | 7 | (INDEXTERMS("animals") NOT INDEXTERMS("humans")) |
| Search Logic | 8 | (1 AND 2 AND 3 AND 4) NOT (5 OR 6 OR 7) |
