# Supplementary material for: Barriers and Facilitators for Population Genetic Screening in Healthy Populations: A Systematic Review
Source: Front Genet. 2022 Jul 4;13:865384. doi: 10.3389/fgene.2022.865384 (PMC9289280; doi:10.3389/fgene.2022.865384)
Supplement: Supplementary file 4 [file Table3.DOCX]

**Appendix C:** PICOS diagram

| Population | Patients, healthcare providers, and the public |
| --- | --- |
| Intervention | Disease area(s), whether population genetic screening was offered, and whether participants met with providers before or after testing |
| Comparator | If applicable |
| Outcomes | Barriers, facilitators, perceptions, effectiveness measures (change in health behavior, results, follow-up, interpretation) |
| Setting | Scale, country, type |
